# Targeting fatty acid synthase sensitizes human nasopharyngeal carcinoma cells to radiation *via* downregulating frizzled class receptor 10

**DOI:** 10.20892/j.issn.2095-3941.2020.0219

**Published:** 2020-08-15

**Authors:** Jiongyu Chen, Fan Zhang, Xiaosha Ren, Yahui Wang, Wenhe Huang, Jianting Zhang, Yukun Cui

**Affiliations:** ^1^Guangdong Provincial Key Laboratory for Breast Cancer Diagnosis and Treatment, Cancer Hospital of Shantou University Medical College, Shantou 515041, China; ^2^Department of Cancer Biology, University of Toledo College of Medicine and Life Sciences, Toledo, OH 43614, USA

**Keywords:** Epigallocatechin gallate, fatty acid synthase, frizzled class receptor 10, nasopharyngeal carcinoma, radioresistance

## Abstract

**Objective:** Our aim was to test the hypothesis that fatty acid synthase (FASN) expression contributes to radioresistance of nasopharyngeal carcinoma (NPC) cells and that inhibiting FASN enhances radiosensitivity.

**Methods:** Targeting FASN using epigallocatechin gallate (EGCG) or RNA interference in NPC cell lines that overexpress endogenous FASN was performed to determine their effects on cellular response to radiation *in vitro* using MTT and colony formation assays, and *in vivo* using xenograft animal models. Western blot, immunohistochemistry, real-time PCR arrays, and real-time RT-PCR were used to determine the relationship between FASN and frizzled class receptor 10 (FZD10) expression. FZD10 knockdown and overexpression were used to determine its role in mediating FASN function in cellular response to radiation. Immunohistochemical staining was used to determine FASN and FZD10 expressions in human NPC tissues, followed by analysis of their association with the overall survival of patients.

**Results:** FASN knockdown or inhibition significantly enhanced radiosensitivity of NPC cells, both *in vitro* and *in vivo*. There was a positive association between FASN and FZD10 expression in NPC cell lines grown as monolayers or xenografts, as well as human tissues. FASN knockdown reduced FZD10 expression, and rescue of FZD10 expression abolished FASN knockdown-induced enhancement of radiosensitivity. FASN and FZD10 were both negatively associated with overall survival of NPC patients.

**Conclusions:** FASN contributes to radioresistance, possibly *via* FZD10 in NPC cells. Both FZD10 and FASN expressions were associated with poor outcomes of NPC patients. EGCG may sensitize radioresistance by inhibiting FASN and may possibly be developed as a radiosensitizer for better treatment of NPCs.

## Introduction

Nasopharyngeal carcinoma (NPC) is distinguished from other head and neck cancers in epidemiology, pathology, and clinical manifestation^1^, and because NPC, especially the moderately/poorly-differentiated tumor, is highly sensitive to radiation, radiotherapy (RT) is a standard treatment of NPC^2^. Unfortunately, radioresistance frequently occurs and no radiosensitizing agents are available to alleviate this clinical issue. Despite increased 5-year survival rates using combined radiochemotherapy^2,3^, the incidence of side effects, namely radiotoxicity and hematological and gastrointestinal toxicities relevant to chemotherapy, increase simultaneously. Hence, novel radiosensitizers that are safe and well tolerated in humans are urgently needed for NPC treatment.

Fatty acid synthase (FASN) has been gaining increased attention as a potential therapeutic target for many cancers^4,5^. Its major function is to catalyze *de novo* synthesis of palmitate by condensing acetyl-CoA and malonyl-CoA with the use of NADPH and ATP. In a well-nourished individual, *de novo* fatty acid synthesis is rarely needed due to the availability of dietary lipids and because FASN expression in normal tissues is low. However, FASN expression has been found to increase in various human cancers to promote cell proliferation^2–4^. Recently, it was found that FASN might contribute to radioresistance, and FASN knockdown activated a caspase cascade in NPC cells^3,5^. Furthermore, FASN has been shown to regulate cellular responses to radiation by controlling repair of radiation-induced DNA damage^6^. Thus, FASN may serve as a target to sensitize NPC to RT.

Epigallocatechin gallate (EGCG), a natural compound from green tea, which has been tested in clinical trials for many cancers, is known to inhibit FASN^7,8^. When compared with other traditional chemotherapies, previous randomized controlled trial studies showed that EGCG was well tolerated by both healthy individuals and cancer patients with a low frequency of adverse effects^9^. In general, EGCG, as a dietary supplement, is considered safe, thus, its use may gain acceptance as a radiosensitizer by targeting FASN.

In this study, we tested the hypothesis that FASN expression contributes to radioresistance of NPC cells and that targeting FASN enhances radiosensitivity. We found that targeting FASN using a RNA interference or EGCG enhanced radiosensitivity of human NPC cells *in vitro* and *in vivo*. Moreover, real-time PCR arrays suggested that knockdown of FASN downregulated the expression of frizzled class receptor 10 (FZD10). In addition, we found that FASN expression significantly associated with FZD10 expression in NPC cell lines and clinical samples. Likewise, rescuing the expression levels of FZD10 in FASN knockdown NPC cells restored radiation resistance, and knockdown of FZD10 enhanced the radiosensitivity of NPC cells. Finally, we found that the expression of FZD10, either alone or in combination with FASN, predicted worse outcomes for NPC patients, and patients with a deficiency of both FZD10 and FASN expressions had the best overall survival. Together these data suggested that FZD10 might mediate FASN endowed radioresistance and that targeting FASN might sensitize human NPC to radiotherapy.

## Materials and methods

### Cell lines and culture conditions

All six human NPC cell lines including CNE1, CNE2, SUNE1, C-661, 5-F8, and HNE1 were generously provided by Dr. Y Cao (Central South University Xiangya Medical College)^10^. These cells were recently tested for mycoplasma and maintained in complete RPMI 1640 medium(Gibco, Thermo Fisher Scientific, San Jose, CA, USA), supplemented with 10% fetal bovine serum (FBS; Biological Industry, Kibbutz Beit Haemek, Israel) at 37 °C, in a 5% CO_2_ incubator. No authentication was done to these cells.

### Establishment of FASN knockdown cells

Two FASN-positive human NPC cell lines, CNE1 and SUNE1, were used to establish stable FASN knockdown cells. Oligonucleotides encoding shRNA targeting human FASN (shFASN)^11^ or with scrambled sequence as a control (shNC) were cloned into the pGPU6/GFP/Neo construct (GenePharmagps, Shanghai, China). The shFASN sequences were as follows.

Forward: 5′-TACGTACTGGCCTACACCCAGA-3′;

reverse: 5′-TGAACTGCTGCACGAAGAAGCATA-3′.

Cells in 6-well plates with 80%–90% confluency were transfected with plasmids encoding shFASN and shNC using Lipofectamine 2000 (Thermo Fisher Scientific) (1.6 μg DNA in 4 uL), followed by selection using 800 μg/mL G418 for 14 days. Pooled clones with FASN knockdown as determined using real-time RT-PCR and Western blot (WB) analyses were collected for further studies.

### FZD10 overexpression in NPC cells

The full-length FZD10 cDNA (GenePharmagps, Shanghai, China) was cloned into a pcDNA3.1 vector, which was transiently transfected into CNE1 cells with stable FASN knockdown or parental CNE2 cells using Lipofectamine 2000™ as described above. At 24 h after transfection, cells were either lysed for WB analyses or subjected to survival analyses.

### FZD10 knockdown in CNE1 cells

Three different siRNAs specific for human FZD10 were synthesized by GenePharma (Shanghai, China) with the following sequences: FZD10-Homo-2091: forward, 5′-GCCGUAGGUUAAAGAAGAATT-3′, reverse, 5′-UUCUUCUUUAACCUACGGCTT-3′; FZD10-Homo- 1819: forward, 5′-GCUCUUCUCUGUGCUGUACTT-3′, reverse, 5′-GUACAGCACAGAGAAGAGCTT-3′; and FZD10- Homo-1581: forward, 5′-CCAUCCUGAUCCUGGUCAUTT-3′, and reverse, 5′-AUGACCAGGAUCAGGAUGGTT-3′ siRNAs. The annealed siRNA duplexes were transfected at 600 pmol per 10 cm plate into CNE1 cells using Lipofectamine RNAiMAX according to the manufacturer’s instructions (Thermo Fisher Scientific). At 48 h after transfection, the cells were subjected to either WB or survival analyses.

### Patients and tumor specimens

Paraffin-embedded archival pathological specimens were available from 122 NPC patients along with complete clinicopathological features. The patients had undergone biopsy without preoperative therapy at the Cancer Hospital of Shantou University Medical College between January 2001 and December 2005. Of this cohort, 99 patients were diagnosed with advanced stages (III/IV), and the rest were at the early stages (I/II). In accordance to the current WHO classification, 110 patients had undifferentiated non-keratinized NPC tumors while the others had differentiated keratinized carcinomas. Lymph node metastasis occurred in 104 NPC patients, who were all free of distant metastasis at initial diagnoses. Clinical tumor stage (TNM stage) was grouped in accordance with the American Joint Committee on Cancer, 6th Edition Cancer Staging Manual (2002). Informed consent was obtained from all patients. This study of tumor samples was approved by the medical ethics committee of the Cancer Hospital of Shantou University Medical College (serial number: 201807).

### Immunohistochemical (IHC) staining

IHC staining of FASN and FZD10 was performed using a standard EnVision complex method^12^. Briefly, tissues were incubated with a monoclonal anti-FASN antibody (1:100 dilution; sc55580; Santa Cruz Biotechnology, Dallas, TX, USA) or rabbit anti-FZD10 polyclonal antibody (1:100 dilution; ab83044; Abcam, Cambridge, UK) and by reaction with an EnVision antibody complex (anti-mouse or rabbit). The final signal was detected using an EnvisionTM Detection kit (ZSGB-BIO, Beijing, China) and 3,3′-diaminobenzidine as the chromogen substrate. Normal IgG was used as a negative control.

The FASN and FZD10 expression levels were estimated from IHC staining by a combination of staining intensity (0, no staining; 1, weak staining; 2, moderate staining; 3, strong staining) and proportion (0, 0% of tumor cells stained; 1, ≤ 10% positive cells; 2, 11%–50% positive cells; 3, 51%–80% positive cells; and 4, > 81% positive cells). If the score of the combination was ≥ 3, it was defined as positive in FASN or FZD10. Cases with scores < 3 were defined as negative. Two pathologists (CJ and XW), blinded to the clinical information of these patients, independently assessed the cellular location and intensity of immunostaining in each section.

### Western blot

Cells were first lysed with a cell lysis buffer containing phenylmethylsulfonyl fluoride (Beyotime, Shanghai, China). Proteins (50 μg) of each cell lysate were then separated by SDS-PAGE and transferred onto a polyvinylidene difluoride membrane followed by blocking with Tris-buffered saline, and incubation at 4 °C overnight with either mouse anti-FASN monoclonal antibody (1:500), rabbit anti-FZD10 monoclonal antibody FZD10 (1:1,000), or anti mouse β-actin antibody (1:3,000; Santa Cruz Biotechnology) in blocking buffer. Following washes with TBST (Tris-buffered saline with Tween 20), the blots were incubated with horseradish peroxidase-labelled anti-rabbit (1:5,000; Novus Biologicals, Littleton, CO, USA) or anti-mouse (1:5,000; Santa Cruz Biotechnology) IgG at room temperature for 2 h, washed with TBST, and followed by signal detection using chemiluminescence.

### Quantitative real-time RT-PCR

Total RNAs were extracted from cells using TRIzol reagent and reverse transcription of purified RNAs was performed using oligo (dT) priming and Superscript III reverse transcriptase according to the manufacturer’s instructions (Invitrogen, Carlsbad, CA, USA). Real-time PCR was performed using the SYBR Premix kit (TaKaRa, Tokyo, Japan) and a Rotor-Gene RG-3000A apparatus (Corbett Research, Brisbane, Australia) with β-actin as an internal control. The primer pairs used for target genes were purchased from Genechem (Shanghai, China) as follows: human FZD10: forward, 5′-CCTCCAAGACTCTGCAGTCC-3′, and reverse, 5′-GACTGGGCAGGGATCTCATA-3′; FASN: forward, 5′-TACGTACTGGCCTACACCCAGA-3′, and reverse, 5′-TGAACTGCTGCACGAAGAAGCATAT-3′; β-actin: forward, 5′-AGCGAGCATCCCCAAGTT-3′, and reverse, 5′-GGGCACGAAGGCTCATCATT-3′.

### MTT survival assay

Cells were seeded in 96-well plates at 1 × 10^4^ cells/well and cultured for 24 h before treatment with EGCG (Amquar, Shanghai, China) or by ionizing radiation using a Varian 600 CD linear accelerator (Varian Medical, Palo Alto, CA, USA). At different times following treatments, the cells were subjected to MTT assays as previously described^6,13^. Briefly, the treated cells were first incubated with 5 mg/mL of the sterile filtered MTT solution (Santa Cruz Biotechnology) in phosphate-buffered saline (PBS) and incubated for 4 h in a moist chamber at 37 °C followed by washing with PBS and incubation with dimethylsulfoxide for 10 min with shaking, followed by determination of solubilized formazan product at *OD*_490nm_ using a microplate reader. The relative inhibition of cell viability was calculated using the formula [1 − (*OD*_treatment_ − *OD*_blank_) / (*OD*_control_ − *OD*_blank_)] × 100%.

### Colony formation assay

Exponentially growing cells were subjected to ionizing radiation treatments at two different doses and resuspended in complete medium for seeding in 6-well plates at a density of 200 cells / well and cultured for 2 weeks. After fixation in paraformaldehyde, the colonies were stained with 0.1% Crystal Violet for 10 min before washing and manual counting. The colony formation efficiency = colony number / 200 cells and the relative colony formation efficiency = efficiency_treated cells_ / efficiency_untreated cells_. The difference of the relative colony formation efficiency was compared between CNE1-shRNA-NC (SUNE1-shRNA-NC) and CNE1-shRNA-FASN (SUNE1-shRNA-FASN) to obtain *P* values.

### NPC xenografts

Twenty nude mice were purchased from Vital River Laboratory Animal Technology (Beijing, China). Each mouse was injected subcutaneously with 5 × 10^6^ CNE1 cells. When the tumors reached approximately 250 mm^3^ at about 10 days after inoculation, the mice were randomized into four groups with five each and treated with 100 μL saline vehicle (control group) or EGCG in saline at 30 μg/kg (EGCG group) *via* intraperitoneal injection daily for 7 days, with 5 Gy single radiation treatment on day 16 (radiation group), or EGCG (daily for 7 days) + 5 Gy radiation (once on day 16) (combination group). The xenograft tumors were measured every 2 days using a digital caliper, and the tumor volume was calculated using the formula: length^2^ × width × 0.5. The final dissected xenograft tumors were fixed with 4% paraformaldehyde and embedded in paraffin for histology and IHC staining analyses. The animal protocol was reviewed and approved by the Animal Ethical and Welfare Committee (AEWC) at Shantou University (serial number: SUMC2018-070).

### EGCG-radiation interaction analysis

The coefficient of drug interaction (CDI) was used to analyze the interactive effects of the EGCG-radiation combination as reported previously^14^ and calculated using the formula: CDI = survival inhibition_combination_ / (survival inhibition_EGCG alone_ × survival inhibition_radiation alone_). A CDI value of < 1, = 1, or > 1 indicated synergistic, additive, or antagonistic interactions, respectively.

### Statistical analysis

Statistical analyses were performed using SPSS statistical software for Windows, version 13.0 (SPSS, Chicago, IL, USA). The comparisons of cell viability, colony formation efficiency, and xenograft tumor size between different treatment groups were calculated using* t*-tests. Survival curves were calculated using the Kaplan-Meier method with a log rank test. The multivariate Cox regression method was used to analyze the effects of FASN / FZD10 expression on overall survival (OS, in months), which was defined as the time from diagnosis to the date of last contact or death. The comparison of 30- or 60-month survival rates between different subpopulations were performed using a *Z*-test. For all tests, a value of *P* < 0.05 was considered significant.

## Results

### FASN knockdown enhanced radiosensitivity of NPC cell lines

Six different human NPC cell lines, including HNE1, SUNE1, 5-F8, C-661, CNE1, and CNE2 along with a human breast cancer cell line control, SKBR-3, which is known to express high level of FASN^15^, were first assessed for FASN expression using WB analysis. As shown in **Supplementary Figure S1A**, FASN expression was abundant in SUNE1, 5-F8, and CNE1, undetectable in C-661 and CNE2, and low in HNE1 cells. To determine the possibility of targeting FASN to increase radiosensitivity, we chose CNE1 and SUNE1cells with high levels of endogenous FASN to evaluate the effect of FASN knockdown (**Supplementary Figure S1B**) on their responses to radiation using the MTT cell viability assay. As shown in **Supplementary Figure S1C**, the survival of both the scrambled control shRNA-transfected cells (CNE1-shRNA-NC and SUNE1-shRNA-NC) and FASN knockdown cells (CNE1-shRNA-FASN and SUNE1-shRNA-FASN) were suppressed by radiation in a time- and dose-dependent manner as determined using MTT assays. However, the radiation-induced inhibition of proliferation of FASN knockdown cells at each time and dose point was significantly less when compared with their respective control cells. Thus, FASN knockdown may sensitize NPC cells to radiation.

To validate the above findings, we next performed colony formation assays. As shown in **Supplementary Figure S1D i–ii**, the colony formation efficiency of CNE1-shRNA-NC control cells declined from 36.0 ± 7.8% to 32.5 ± 3.0% and 11.3 ± 1.5% after treatment with 2 and 5 Gy radiation, respectively. However, the colony formation efficiency of CNE1-shRNA-FASN cells showed a more substantial decrease, dropping from 22.3 ± 2.5% to 13.0 ± 2.0% (*P* = 0.002) and 4.2 ± 1.5% (*P* = 0.006) after treatment with 2 and 5 Gy radiation, respectively. In a similar manner, the colony formation efficiency of the SUNE1-shRNA-NC control cells barely changed following exposure to 2 Gy radiation and decreased from 41.7 ± 4.5% to 29.0 ± 4.0% following exposure to 5 Gy radiation, while the colony formation efficiency of SUNE1-shRNA-FASN cells declined from 24.8 ± 4.0% to 18.3 ± 3.8% (*P* = 0.008) and 14.5 ± 2.6% (*P* = 0.030) after exposure to 2 and 5 Gy radiation, respectively (**Supplementary Figure S1D i and iii**). These findings suggested that FASN knockdown sensitized NPC cells to radiation.

### FASN might be a therapeutic target of EGCG in NPC cells

To determine if inhibiting FASN function could also sensitize NPC cells to radiation, we chose to use EGCG, which has previously been shown to inhibit FASN^7,8^ and is well tolerated in humans for potential clinical translation. However, because EGCG has also been shown to inhibit other proteins in addition to FASN, we first determined the selectivity of EGCG to FASN in NPC cells. CNE1 and SUNE1 cells with or without stable FASN knockdown were treated with increasing concentrations of EGCG followed by analyses using MTT assays. As shown in **Supplementary Figure S2A**, 24 h of EGCG treatment at low concentrations (25 and 50 μg/mL) caused little inhibition to NPC cells with FASN knockdown (CNE1-shRNA-FASN and SUNE1-shRNA-FASN), while significant inhibition was observed with the scrambled shRNA-transfected control cells. Treatment with higher concentrations (100 and 150 μg/mL) of EGCG for 24 h resulted in significant, but less inhibition of survival with the CNE1-shRNA-FASN and SUNE1-shRNA-FASN than the control CNE1-ShRNA-NC and SUNE1-ShRNA-NC cells. Treatment for 48 h with EGCG at 25, 50, 100, and 150 μg/mL resulted in 8.35%, 24.36%, 76.00%, and 91.60% inhibition for CNE1-shRNA-NC cells, respectively, while only 0.05% (*P* < 0.001), 0.71% (*P* < 0.001), 45.18% (*P* < 0.001), and 85.81% (*P* < 0.001) inhibitions were observed for CNE1-shRNA-FASN cells (**Supplementary Figure S2A**). Similarly, treatment of SUNE1-shRNA-NC cells for 48 h with increasing EGCG concentrations (25, 50, 100, and 150 μg/mL) resulted in more dramatic increasing inhibition (20.38%, 38.29%, 62.50%, and 90.14%, respectively) than that of SUNE1-shRNA-FASN cells (0.18%, *P* < 0.001; 26.10%, *P* < 0.001; 56.80%, *P* < 0.001, and 87.66%, *P* < 0.001, respectively) (**Supplementary Figure S2A**).

The colony formation assay was further conducted to assess the cell viability at the concentrations of 25 and 50 μg/mL EGCG. Compared with cells cultured in EGCG-free medium, the colony formation efficiency of CNE1-shRNA-NC decreased from 38.0 ± 8.7% separately to 22.5 ± 5.0% and 11.7 ± 4.2%, while that of CNE1-shRNA-FASN merely declined from 21.8 ± 2.1% to 18.8 ± 2.5% (*P*<0.001) and 15.0 ± 2.0% (*P* = 0.0012, respectively (**Supplementary Figure S2B**). Likewise, the colony formation efficiency of EGCG-supplied SUNE1-shRNA-NC (declining from 40.3 ± 3.8% to 21.0 ± 2.0% and 10.8 ± 1.5%) was significantly lower than that of EGCG-supplied SUNE1-shRNA-FASN (only declining to from 24.2 ± 4.0% to 21.0 ± 2.0% and 15.8 ± 3.5%, both *P* values <0.001) (**Supplementary Figure S2B**). These findings suggested that EGCG at low concentrations may be selective to FASN, and that FASN may mediate EGCG effects on NPC cell survival.

### EGCG-radiation interaction analysis

**Figure 1:**
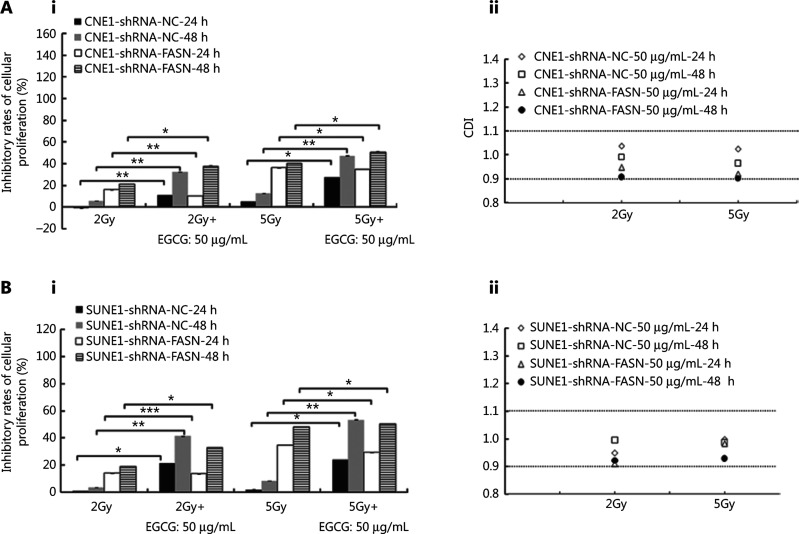
Epigallocatechin gallate (EGCG) additively enhances the sensitivity of nasopharyngeal carcinoma cells to radiation *in vitro*. A–B, The effect of 50 ?g/mL EGCG on radiation-induced inhibition in proliferation of CNE1 (A) and SUNE1 (B) cells with or without fatty acid synthase knockdown. The panels Aii and Bii show coefficients of drug interaction between EGCG and different dosages of radiation at different time points.

We next examined whether radiation interacted with low concentrations of EGCG in combination treatments of NPC cells with or without FASN knockdown. As shown in **Figure 1Ai**, 50 μg/mL EGCG significantly enhanced the 2 Gy radiation-induced inhibition of control CNE1-shRNA-NC cell survival by an average of ˜ 5.7-fold, and enhanced the 5 Gy radiation-induced inhibition by ˜ 3.8-fold. Similar results were found with SUNE1-shRNA-NC cells (**Figure 1Bi**). However, 50 μg/mL EGCG resulted in little enhancement of 2 Gy and 5 Gy radiation-induced inhibition of survival of CNE1-shRNA-FASN (1.8- and 1.3-fold, respectively) and SUNE1-shRNA-FASN (1.7- and 1.0-fold, respectively) cells (**Figure 1Ai** and **Figure 1Bi**). These results suggested that EGCG may affect radiation treatment, possibly by inhibiting FASN.

To further understand the EGCG-radiation interaction, CDIs were calculated. As shown in **Figure 1Aii and Figure 1Bii**, the CDI values varied from 0.90 to 1.04 for different combinations in different cells, suggesting that EGCG and radiation may have an additive relationship.

### EGCG increased the radiation sensitivity of NPC tumors *in vivo*

**Figure 2:**
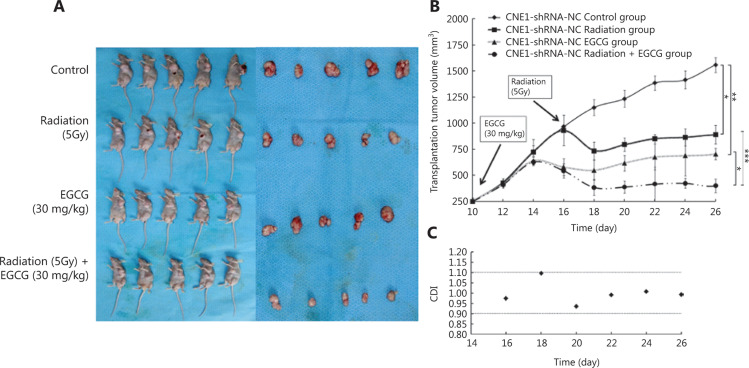
Epigallocatechin gallate (EGCG) additively enhances the sensitivity of nasopharyngeal carcinoma cells to radiation *in vivo*. A, Nude mice with tumors and gross anatomy of dissected xenograft tumors of CNE1-shRNA-NC cells at the conclusion of treatment. B, Growth curve of CNE1-shRNA-NC cell xenograft tumors in different groups of treatments. C, Coefficients of drug interaction between sequential administration of EGCG and a single dose of radiation at different time points. *P < 0.05; **P < 0.01; ***P < 0.001.

We next tested the combined effect of EGCG with radiation on xenograft tumors *in vivo* using CNE1-shRNA-NC cell injections (CNE1-shRNA-FASN cells could not form xenografted tumors). As shown in **Figure 2A–B**, tumors of the control group exhibited sustained growth during the period of study with an 11.5-fold increase in tumor volume from days 10 to 26. Tumors of the 5 Gy radiation alone and EGCG alone treatment groups showed 3.7- and 3.2-fold increases in tumor volumes, respectively, which represented significant reductions (*P* = 0.007 and *P* < 0.001, respectively) in tumor volumes compared to the control-treated group. The combination of EGCG with radiation further repressed tumor growth with only a 2.2-fold increase in tumor volume during the period of the study, a significant (*P* < 0.001) reduction compared with EGCG or radiation alone treatment. It was also noteworthy that CNE1-shRNA-FASN cells could not form xenograft tumors (data not shown), consistent with the finding that EGCG inhibited CNE1-shRNA-NC xenograft tumor growth. Furthermore, CDIs of EGCG and radiation combination were calculated to range from 0.94 to 1.10 (**Figure 2C**), consistent with the *in vitro* findings. Thus, EGCG and radiation may additively inhibit NPC tumor growth.

### FZD10 expression was regulated by FASN and radiation

**Figure 3:**
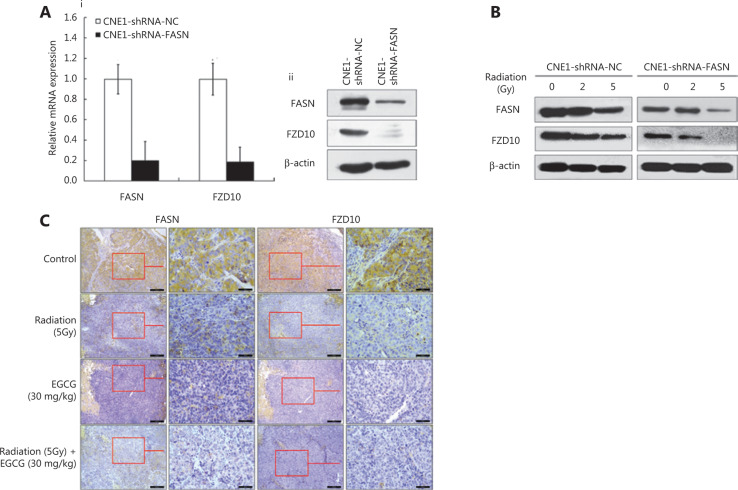
Regulation of FZD10 expression by fatty acid synthase (FASN) and radiation. A, Effect of FASN knockdown on frizzled class receptor 10 (FZD10) expression in CNE1 cells as determined using RT-PCR (panel i) and WB (panel ii). B, Effect of radiation on FASN and FZD10 expression in CNE 1 cells with or without FASN knockdown. C, Effect of radiation and epigallocatechin gallate on FASN and FZD10 expressions in xenograft tumors *in vivo* as determined using immunohistochemical staining. The right panels are magnified (400× magnification) from the left panels (100× magnification) for both FASN and FZD10.

To identify the underlying mechanism of FASN overexpression associated with NPC cell resistance to radiotherapy, we performed a real-time PCR array to examine the impact of FASN on the expression of Wnt signaling effectors, because previous studies suggested a role of FASN knockdown in the attenuated Wnt signaling pathway^16,17^ (**Supplementary Table S1**). Among these effectors, expression of FZD10, which has been proposed as a promising target of radioimmune therapy for synovial sarcoma^18,19^, was almost eliminated by knockdown of FASN in NPC cells. This observation was validated using real-time RT-PCR and immunoblotting as shown in **Figure 3A**. FZD10 mRNA was significantly decreased by ˜80% in CNE1-shRNA-FASN cells compared to CNE1-shNC-FASNcells. A reduction in FZD10 protein level was also observed in CNE1-shRNA-FASN cells compared to CNE1-shRNA-NC cells. Furthermore, both FASN and FZD10 were dose-dependently reduced by radiation in both CNE1-shRNA-NC and CNE1-shRNA-FASN cells (**Figure 3B**). Interestingly, manipulating FZD10 expression did not alter FASN expression (see below). Together, these results suggested that FASN regulated FZD10 expression, but not vice versa.

We also examined whether inhibiting the expression or activity of FASN, as well as radiation treatment, could reduce the expression of FZD10 *in vivo* by determining FASN and FZD10 expressions in the 20 CNE1-shRNA-NC xenograft tumors from the above studies in **Figure 2**, using immunohistochemistry. As shown in **Figure 3C** and **Supplementary Tables S2** and **S3**, EGCG, radiation, or the combination reduced the expression of both FASN and FZD10, when compared to that in the control group. Thus, FASN likely conferred NPC radioresistance *via* regulating the expression of FZD10 both *in vitro* and *in vivo*.

### FZD10 was associated with FASN in NPC cell lines and human NPC tumors

**Figure 4:**
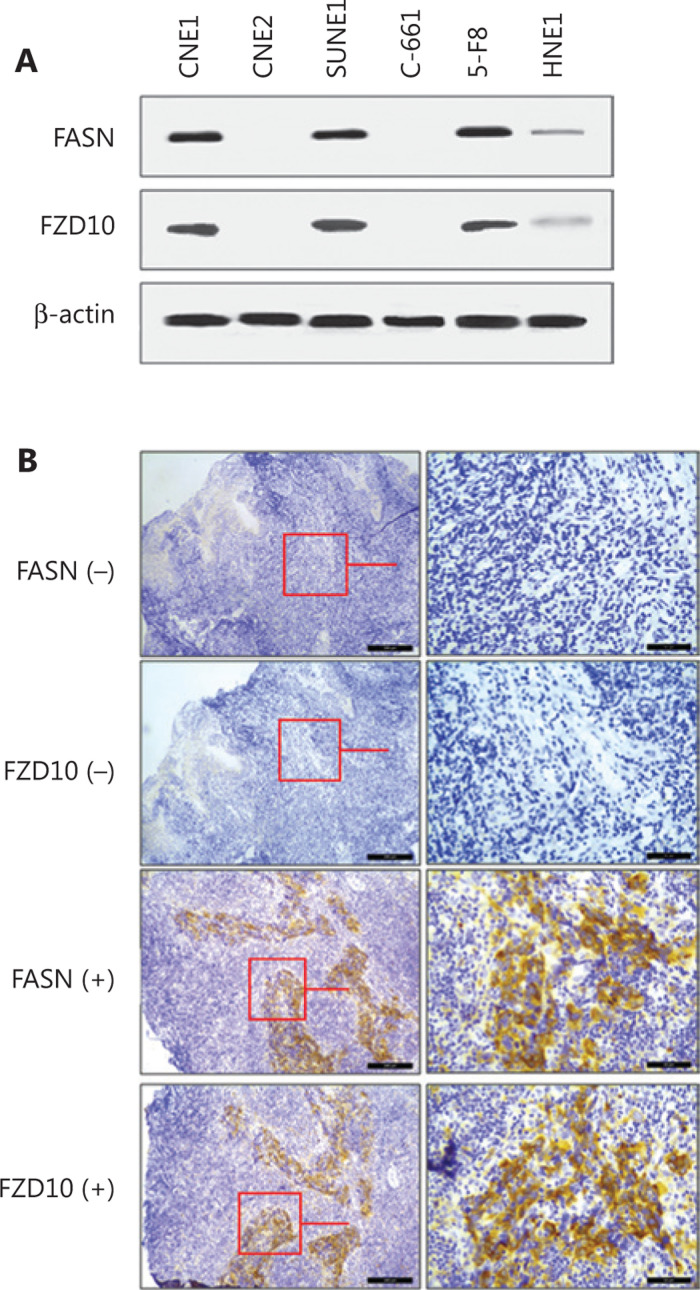
Co-expression of fatty acid synthase (FASN) and frizzled class receptor 10 (FZD10) in human nasopharyngeal carcinoma (NPC) cell lines and tumor samples. A, western blot analysis of FZD10 and FASN in different NPC cell lines. β-Actin was used as a loading control. B, Representative immunohistochemical staining of FASN and FZD10 in human NPC tumors (*n* = 122).

As previously mentioned, FZD10 might also be used as a target to enhance RT efficacy in the treatment of NPC. To test this possibility, we first determined FZD10 expression in human NPC cell lines and tumors using WB and immunohistochemistry, respectively. Surprisingly, we found that FASN and FZD10 were co-expressed in 6 human NPC cell lines (**Figure 4A**) and in 122 human NPC tumors (**Figure 4B** and **Supplementary Tables S4, S5**). Approximately 86% (62 of 72) of FASN positive tumors were also FZD10 positive compared with ˜ 66% (81/122) of all tumors being FZD10 positive (*P* = 0.003). Approximately 62% (31/50) of FASN-negative tumors were also FZD10 deficient compared with only ˜ 34% (41/122) of FZD10-deficient tumors in all samples (*P* = 0.001). In a similar manner, the rate of positive FASN expressions in FZD10 positive tumors (˜ 77%, 62/81) was significantly (*P* = 0.010) higher than that in all samples (˜ 59%, 72/122) and the rate of FASN deficiency in FZD10 negative tumors (˜ 76%, 31/41) was also significantly (*P* < 0.001) higher than that in all tumors (˜ 41%, 50/122). These findings suggested that FASN and FZD10 expression may be positively associated with each other in human NPC cell lines and tumors.

### FZD10 contributed to and mediated FASN function in radiation resistance

**Figure 5:**
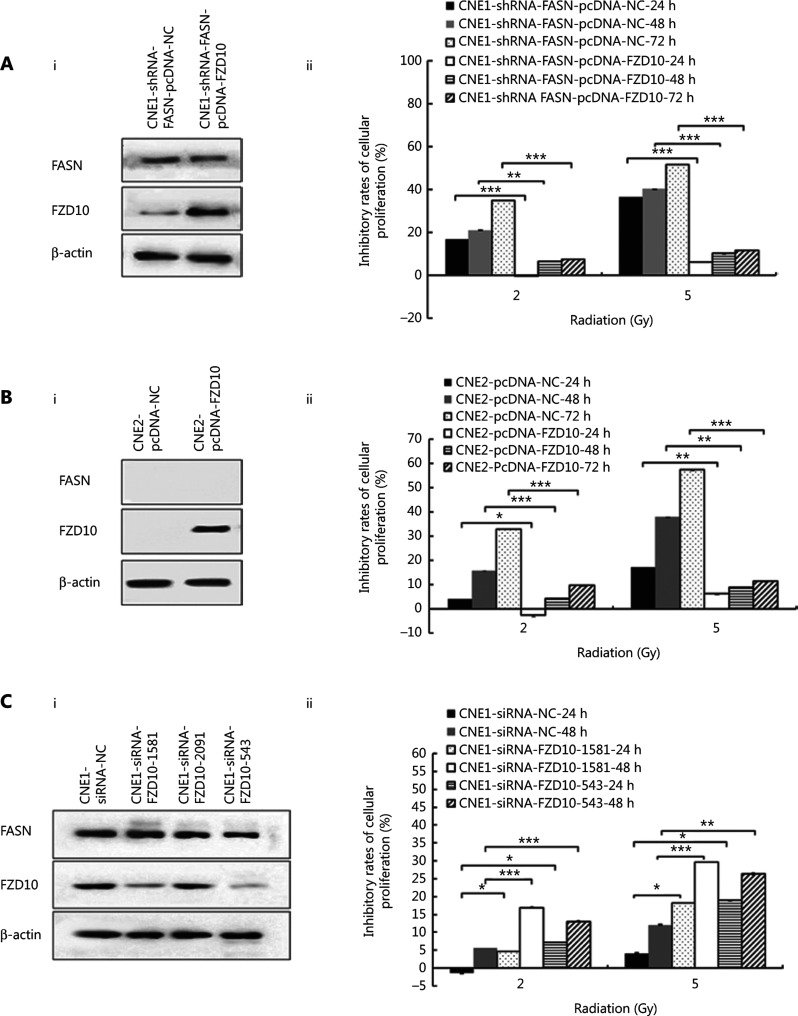
The role of frizzled class receptor 10 (FZD10) in nasopharyngeal carcinoma cell response to radiation. A, FZD10 overexpression rescues fatty acid synthase knockdown-induced downregulation in FZD10 expression and cellular resistance to radiation. B, FZD10 overexpression confers radiation resistance in CNE2 cells. C, FZD10 knockdown using siRNA augments radiosensitivity in CNE1 cells. Panel i shows WB analysis and panel ii shows MTT survival assays after manipulation ofFZD10 expression. *P < 0.05; **P < 0.01; ***P < 0.001.

To investigate whether FZD10 mediated FASN-induced radiation resistance in NPC cells, we transiently overexpressed ectopic FZD10 (pcDNA3.1-FZD10) in CNE1-shRNA-FASN cells to rescue the expression of FZD10, which was reduced by FASN knockdown. We also performed similar expression studies using CNE2 cells deficient in both FASN and FZD10. As shown in **Figure 5Ai** and **5Bi**, ectopic FZD10 expression in CNE1-shRNA-FASN or the parental CNE2 cells did not affect the expression of FASN. Knocking down FZD10 expression in the parental CNE1 cells also did not change FASN expression (**Figure 5Ci**). Thus, FZD10 did not regulate FASN expression.

Next, we performed MTT assays to determine whether ectopic FZD10 expression affected radiation responses. Notably, FZD10 overexpression in both CNE1-shRNA-FASN and CNE2 cells significantly abrogated radiation inhibition of cell survival (**Figure 5Aii** and **5Bii**), whereas FZD10 knockdown in CNE1 cells enhanced the radiation inhibition of cell proliferation (**Figure 5Cii**). Together, these data suggested that FASN-induced NPC resistance to radiation may be partially mediated by FZD10.

### FASN and FZD10 deficiencies were associated with favorable outcomes for NPC patients

**Figure 6:**
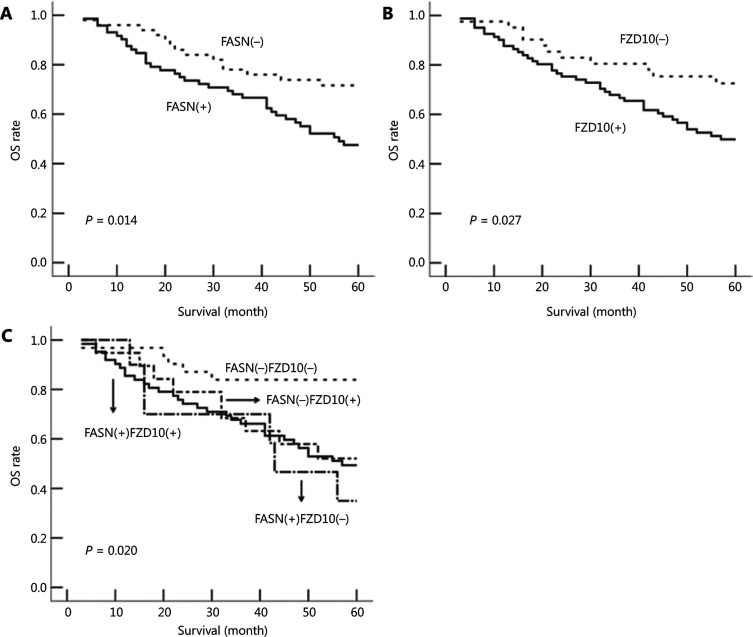
Overall survival analyses of nasopharyngeal carcinoma patients. A-B, Comparison of overall survival between patients with high (*N* = 72) and low fatty acid synthase (FASN) levels (log-rank, *P* = 0.014, *N* = 50, A) and between patients with high (*N* = 81) and low frizzled class receptor 10 (FZD10) levels (log-rank, *P* = 0.027,*N* = 41, B). C, Comparison between patients in four groups with different combinations of FASN and FZD10 expression status (log-rank, *P* = 0.020; FASN+FZD10+: *N* = 62; FASN+FZD10−: *N* = 10; FASN-FZD10+: *N* = 19; FASN-FZD10−: *N*= 31).

Previously, it has been reported that FASN overexpression was associated with poor outcomes in NPC patients^5^. It was of interest to determine if the expression status of FZD10 and/or FASN was associated with the OS of NPC patients, as determined using univariate and multivariate analyses with the Kaplan-Meier method and Cox regression, as well as comparison of OS using *Z*-tests. As shown in **Figure 6A**, OS of patients in the FASN(+) group was significantly lower than that in the FASN(−) group (logrank: *P* = 0.014). Multivariate analyses indicated that FASN was an independent unfavorable prognostic factor for NPC patients after being adjusted for age, sex, and lymph node metastasis [hazard ratio (HR): 1.969, 95% confidence interval (CI): 1.061–3.654; *P* = 0.032) (**Supplementary Table S6**), consistent with a previous report^5^. As expected, a significant difference in OS was also observed between the FZD10(+) and FZD10(−) groups (logrank: *P* = 0.027) (**Figure 6B**). However, no statistical significance was observed between these two groups following multivariate analysis, but a near significant difference was observed (HR: 1.949; 95%CI: 0.990–3.837; *P* = 0.054). We further divided these patients into four groups by combining the expressions of FASN and FZD10 [FASN(+)FZD10(+), FASN(−)FZD10(+), FASN(+)FZD10(−), and FASN(−)FZD10(−)] and compared their OS differences. As shown in **Figure 6C** and **Supplementary Table S6**, OS of patients in the FASN(−)/FZD10 (−) group was significantly better than that of the other three groups. However, we did not observe significant differences in OS among the other three groups [FASN(+)FZD10(+), FASN(−)FZD10(+), and FASN(+)FZD10(−)]. Similar results were obtained after multivariate Cox analyses (**Supplementary Table S6**).

## Discussion

**Figure 7:**
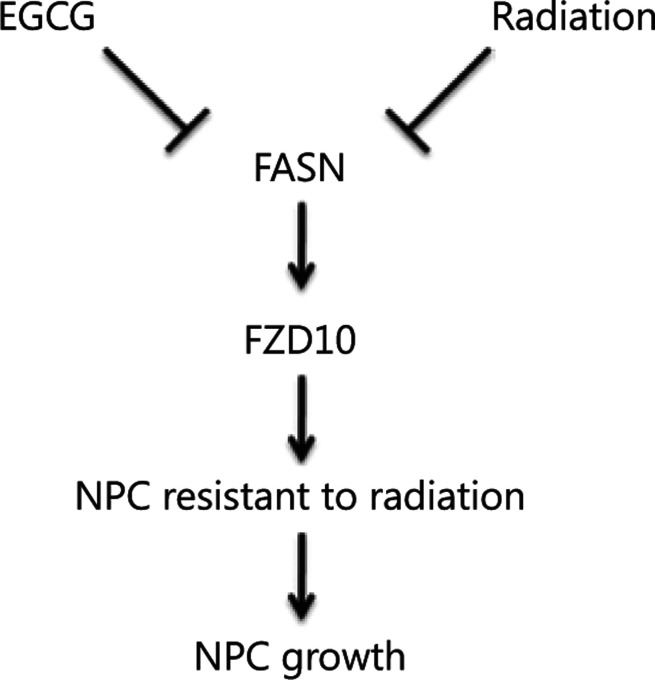
A model scheme delineating a potential mechanism underlying fatty acid synthase-mediated nasopharyngeal carcinoma radioresistance.

In this study, we showed that FASN may contribute to radiation resistance and poor outcomes of NPC patients. Inhibiting FASN may enhance NPC cell sensitivity to radiation. We also showed that FZD10 may be a downstream target of FASN and could mediate FASN function in radiation resistance. EGCG at low concentrations appeared to be selective for FASN in NPC cells and additively sensitized NPC cells to radiation, possibly *via* inhibiting FASN (**Figure 7**). Thus, EGCG may be developed and tested as a radiosensitizing agent for NPC treatments.

It is well accepted that FASN expression contributes to drug resistance, which in turn may cause disease relapse and ultimate failure in chemotherapy. The function of FASN in drug resistance has been shown in pancreatic cancer cells resistant to gemcitabine^20^ and luminal A breast cancer cells (MCF7) resistant to adriamycin, Her2-overexpressing breast cancer cell lines (SK-Br3) resistant to docetaxel^13^, and ovarian cancer cells resistant to Herceptin^21^. Although knowledge regarding FASN in radiation resistance is limited, it has been reported that FASN overexpression could render pancreatic and breast cancer cells resistant to radiation^6,22^. FASN was upregulated in the radiation resistant head and neck squamous carcinoma cell line, rSCC-61, compared to the parental SCC-61 cells^23,24^. Together with previous studies, our results showed that FASN overexpression likely contributed to radioresistance in NPC cells.

While a detailed mechanism of FASN function in drug and radiation resistance remains elusive, recent studies suggested that FASN might function to prevent drug and radiation-induced apoptosis *via* regulating ceramide production^25^. FASN has recently been found to regulate the repair of DNA damage induced by radiation or drugs *via* regulating PARP1 expression^26^. It has also been proposed that, instead of energy storage in the form of triacylglycerol, newly synthesized lipids produced by FASN in cancer cells preferentially became phospholipids, which were involved in cell signaling or cell membrane composition that might contribute to resistance^20^. Other pathways such as the PI3K/Akt pathway might also mediate FASN contributions to drug and radiation resistance^27^.

The Wnt/β-catenin pathway has been suggested to mediate radioresistance in several cancers^28^. As a secreted glycoprotein, Wnt requires covalent modification with a cis-unsaturated fatty acyl group at a conserved serine residue for secretion and activity, and initiates signaling through binding to cell-surface FZD receptors^29^, which could be one mechanism underlying FASN regulated Wnt signaling that might confer cancer cell radioresistance. Here, we observed that FASN-upregulated FZD10 also participated in and mediated FASN functions in radiation resistance in NPC cells; it was therefore of interest to address whether Wnt was essential for FZD10-conferred radioresistance of NPC cell, but this was out of the scope of the current study. Although both FASN and FZD10 could independently predict the outcome of NPC patients, NPC patients lacking both FASN and FZD10 had the best OS when compared to other groups with different combinations of expression profiles of FASN and FZD10.

## Conclusions

We found that FASN and FZD10 were prognostic predictors of NPC cancer outcomes, and that inhibiting FASN may enhance NPC cell responsiveness to radiation, probably *via* downregulating the expression of FZD10. Therefore, a combination of RT and FASN-targeted therapies may provide a new management approach for FASN-positive NPC patients. To this end, EGCG may be tested and developed as a therapeutic approach in combination with RT for better treatment of NPC patients.
