# Supplementary material for: Targeting fatty acid synthase sensitizes human nasopharyngeal carcinoma cells to radiation via downregulating frizzled class receptor 10
Source: Cancer Biol Med. 2020 Aug 15;17(3):740–52. doi: 10.20892/j.issn.2095-3941.2020.0219 (PMC7476091; doi:10.20892/j.issn.2095-3941.2020.0219)
Supplement: Supplementary file 1 [file cbm-17-740-s001.pdf]

Supplementary materials

Supplementary Table S1 The expression of genes in the Wnt/β-catenin pathway at the transcriptional level in CNE1-shRNA-FASN and its parental cell line (CNE1)

Well	Fluor	Content	Target	Sample[b]	Threshold cycle (C(t))
A01	FAM	Unkn	18S	1	14.84
A02	FAM	Unkn	GAPDH	1	19.52
A03	FAM	Unkn	HPRT1	1	26.82
A04	FAM	Unkn	GUSB	1	27.06
A05	FAM	Unkn	APC	1	27.10
A06	FAM	Unkn	AXIN1	1	27.88
A07	FAM	Unkn	AXIN2	1	32.27
A08	FAM	Unkn	BTRC	1	29.31
A09	FAM	Unkn	CSNK1A1	1	28.04
A10	FAM	Unkn	CSNK1D	1	26.30
A11	FAM	Unkn	CSNK1G1	1	29.64
A12	FAM	Unkn	CSNK1G2	1	39.49
B01	FAM	Unkn	CSNK1G3	1	27.85
B02	FAM	Unkn	CSNK2A1	1	25.84
B03	FAM	Unkn	CSNK2A2	1	29.28
B04	FAM	Unkn	CSNK2B	1	22.94
B05	FAM	Unkn	CTNNB1	1	25.86
B06	FAM	Unkn	CTNNBIP1	1	29.79
B07	FAM	Unkn	CXXC4	1	–[a]
B08	FAM	Unkn	DACT1	1	37.52
B09	FAM	Unkn	DKK1	1	28.52
B10	FAM	Unkn	DKK2	1	–[a]
B11	FAM	Unkn	DKK3	1	35.00
B12	FAM	Unkn	DKK4	1	–[a]
C01	FAM	Unkn	DVL1	1	31.50
C02	FAM	Unkn	DVL2	1	29.35
C03	FAM	Unkn	DVL3	1	25.52
C04	FAM	Unkn	EP300	1	28.06
C05	FAM	Unkn	FBXW11	1	29.46
C06	FAM	Unkn	FGF4	1	–[a]
C07	FAM	Unkn	FOXN1	1	37.35
C08	FAM	Unkn	FRAT1	1	31.68

Continued

Well	Fluor	Content	Target	Sample[b]	Threshold cycle (C(t))
C09	FAM	Unkn	FRAT2	1	26.85
C10	FAM	Unkn	FRZB	1	–[a]
C11	FAM	Unkn	FZD1	1	31.11
C12	FAM	Unkn	FZD10	1	32.76
D01	FAM	Unkn	FZD2	1	30.06
D02	FAM	Unkn	FZD3	1	30.52
D03	FAM	Unkn	FZD4	1	28.18
D04	FAM	Unkn	FZD6	1	25.94
D05	FAM	Unkn	FZD7	1	30.19
D06	FAM	Unkn	FZD8	1	31.62
D07	FAM	Unkn	FZD9	1	35.70
D08	FAM	Unkn	GSK3A	1	28.76
D09	FAM	Unkn	GSK3B	1	26.60
D10	FAM	Unkn	KREMEN1	1	29.17
D11	FAM	Unkn	KREMEN2	1	35.11
D12	FAM	Unkn	LEF1	1	28.62
E01	FAM	Unkn	LRP5	1	25.48
E02	FAM	Unkn	LRP6	1	27.65
E03	FAM	Unkn	MYC	1	23.54
E04	FAM	Unkn	NKD1	1	33.06
E05	FAM	Unkn	NLK	1	31.40
E06	FAM	Unkn	CBY1	1	29.03
E07	FAM	Unkn	PITX2	1	38.53
E08	FAM	Unkn	PORCN	1	28.53
E09	FAM	Unkn	PPP2CA	1	25.51
E10	FAM	Unkn	PPP2R1A	1	26.37
E11	FAM	Unkn	PYGO1	1	–[a]
E12	FAM	Unkn	PYGO2	1	27.92
F01	FAM	Unkn	RHOU	1	35.92
F02	FAM	Unkn	SENP2	1	28.01
F03	FAM	Unkn	SFRP1	1	37.59
F04	FAM	Unkn	SFRP2	1	–[a]
F05	FAM	Unkn	SFRP4	1	–[a]

Supplementary Table S1 Continued

Well	Fluor	Content	Target	Sample[b]	Threshold cycle (C(t))
F06	FAM	Unkn	SFRP5	1	–[a]
F07	FAM	Unkn	SLC9A3R1	1	30.43
F08	FAM	Unkn	TCF7	1	32.23
F09	FAM	Unkn	TCF7L1	1	32.42
F10	FAM	Unkn	TCF7L2	1	27.29
F11	FAM	Unkn	TLE1	1	29.99
F12	FAM	Unkn	TLE2	1	33.29
G01	FAM	Unkn	TLE3	1	26.13
G02	FAM	Unkn	TLE4	1	32.37
G03	FAM	Unkn	TLE6	1	35.79
G04	FAM	Unkn	WIF1	1	–[a]
G05	FAM	Unkn	WISP1	1	–[a]
G06	FAM	Unkn	WNT1	1	–a
G07	FAM	Unkn	WNT10A	1	38.10
G08	FAM	Unkn	WNT10B	1	31.89
G09	FAM	Unkn	WNT11	1	32.22
G10	FAM	Unkn	WNT16	1	37.70
G11	FAM	Unkn	WNT2	1	–[a]
G12	FAM	Unkn	WNT2B	1	37.35
H01	FAM	Unkn	WNT3	1	–[a]
H02	FAM	Unkn	WNT3A	1	37.98
H03	FAM	Unkn	WNT4	1	36.54
H04	FAM	Unkn	WNT5A	1	28.58
H05	FAM	Unkn	WNT5B	1	34.74
H06	FAM	Unkn	WNT6	1	38.34
H07	FAM	Unkn	WNT7A	1	34.56
H08	FAM	Unkn	WNT7B	1	32.94
H09	FAM	Unkn	WNT8A	1	–[a]
H10	FAM	Unkn	WNT8B	1	–[a]
H11	FAM	Unkn	WNT9A	1	39.41
H12	FAM	Unkn	WNT9B	1	34.42
A01	FAM	Unkn	18S	2	18.65
A02	FAM	Unkn	GAPDH	2	22.82
A03	FAM	Unkn	HPRT1	2	29.31
A04	FAM	Unkn	GUSB	2	29.04
A05	FAM	Unkn	APC	2	31.51
A06	FAM	Unkn	AXIN1	2	30.56
A07	FAM	Unkn	AXIN2	2	36.12
A08	FAM	Unkn	BTRC	2	31.02
A09	FAM	Unkn	CSNK1A1	2	35.76
A10	FAM	Unkn	CSNK1D	2	–[a]
A11	FAM	Unkn	CSNK1G1	2	–[a]
A12	FAM	Unkn	CSNK1G2	2	–[a]
B01	FAM	Unkn	CSNK1G3	2	31.14
B02	FAM	Unkn	CSNK2A1	2	29.13
B03	FAM	Unkn	CSNK2A2	2	31.22
B04	FAM	Unkn	CSNK2B	2	26.20
B05	FAM	Unkn	CTNNB1	2	27.81
B06	FAM	Unkn	CTNNBIP1	2	30.48
B07	FAM	Unkn	CXXC4	2	37.94
B08	FAM	Unkn	DACT1	2	36.86
B09	FAM	Unkn	DKK1	2	29.80
B10	FAM	Unkn	DKK2	2	–[a]
B11	FAM	Unkn	DKK3	2	36.61
B12	FAM	Unkn	DKK4	2	–[a]
C01	FAM	Unkn	DVL1	2	30.15
C02	FAM	Unkn	DVL2	2	32.79
C03	FAM	Unkn	DVL3	2	27.74
C04	FAM	Unkn	EP300	2	31.65
C05	FAM	Unkn	FBXW11	2	32.05
C06	FAM	Unkn	FGF4	2	–[a]
C07	FAM	Unkn	FOXN1	2	43.76
C08	FAM	Unkn	FRAT1	2	33.74
C09	FAM	Unkn	FRAT2	2	29.92
C10	FAM	Unkn	FRZB	2	–[a]
C11	FAM	Unkn	FZD1	2	32.02
C12	FAM	Unkn	FZD10	2	–[a]
D01	FAM	Unkn	FZD2	2	32.14
D02	FAM	Unkn	FZD3	2	32.85
D03	FAM	Unkn	FZD4	2	30.40

Supplementary Table S1 Continued

Well	Fluor	Content	Target	Sample[b]	Threshold cycle (C(t))
D04	FAM	Unkn	FZD6	2	28.19
D05	FAM	Unkn	FZD7	2	33.17
D06	FAM	Unkn	FZD8	2	32.01
D07	FAM	Unkn	FZD9	2	36.73
D08	FAM	Unkn	GSK3A	2	29.28
D09	FAM	Unkn	GSK3B	2	28.89
D10	FAM	Unkn	KREMEN1	2	33.97
D11	FAM	Unkn	KREMEN2	2	45.79
D12	FAM	Unkn	LEF1	2	–[a]
E01	FAM	Unkn	LRP5	2	28.84
E02	FAM	Unkn	LRP6	2	31.59
E03	FAM	Unkn	MYC	2	26.12
E04	FAM	Unkn	NKD1	2	35
E05	FAM	Unkn	NLK	2	33.35
E06	FAM	Unkn	CBY1	2	31.47
E07	FAM	Unkn	PITX2	2	40.02
E08	FAM	Unkn	PORCN	2	30.99
E09	FAM	Unkn	PPP2CA	2	26.98
E10	FAM	Unkn	PPP2R1A	2	26.90
E11	FAM	Unkn	PYGO1	2	–[a]
E12	FAM	Unkn	PYGO2	2	–[a]
F01	FAM	Unkn	RHOU	2	36.96
F02	FAM	Unkn	SENP2	2	29.36
F03	FAM	Unkn	SFRP1	2	–[a]
F04	FAM	Unkn	SFRP2	2	40.50
F05	FAM	Unkn	SFRP4	2	–[a]
F06	FAM	Unkn	SFRP5	2	–[a]
F07	FAM	Unkn	SLC9A3R1	2	32.83
F08	FAM	Unkn	TCF7	2	33.88
F09	FAM	Unkn	TCF7L1	2	34.59
F10	FAM	Unkn	TCF7L2	2	31.93
F11	FAM	Unkn	TLE1	2	–[a]
F12	FAM	Unkn	TLE2	2	–[a]
G01	FAM	Unkn	TLE3	2	28.27
G02	FAM	Unkn	TLE4	2	33.87
G03	FAM	Unkn	TLE6	2	35.48
G04	FAM	Unkn	WIF1	2	–[a]
G05	FAM	Unkn	WISP1	2	–[a]
G06	FAM	Unkn	WNT1	2	40.11
G07	FAM	Unkn	WNT10A	2	36.42
G08	FAM	Unkn	WNT10B	2	31.77
G09	FAM	Unkn	WNT11	2	34.89
G10	FAM	Unkn	WNT16	2	40.72
G11	FAM	Unkn	WNT2	2	–[a]
G12	FAM	Unkn	WNT2B	2	–[a]
H01	FAM	Unkn	WNT3	2	33.86
H02	FAM	Unkn	WNT3A	2	–[a]
H03	FAM	Unkn	WNT4	2	36.55
H04	FAM	Unkn	WNT5A	2	32.58
H05	FAM	Unkn	WNT5B	2	36.38
H06	FAM	Unkn	WNT6	2	37.63
H07	FAM	Unkn	WNT7A	2	33.55
H08	FAM	Unkn	WNT7B	2	32.72
H09	FAM	Unkn	WNT8A	2	37.86
H10	FAM	Unkn	WNT8B	2	–[a]
H11	FAM	Unkn	WNT9A	2	45.16
H12	FAM	Unkn	WNT9B	2	43.62

[a]Representing those genes without amplification in the samples; [b]Sample 2 and Sample 1 separately referred to CNE1 with and without downregulating fatty acid synthase using a short hairpin RNA.

Supplementary Table S2 Expression status of fatty acid synthase (FASN) and frizzled class receptor 10 (FZD10) in xenografts of mice

# of mouse[†]	Control		Radiation (5 Gy)		EGCG (30 mg/kg)		Radiation (5 Gy) and EGCG (30 mg/kg)	
	FASN	FZD10	FASN	FZD10	FASN	FZD10	FASN	FZD10
1	4	4	1	1	1	1	0	0
2	4	4	0	0	0	0	0	0
3	2	2	0	0	0	0	0	0
4	4	4	0	0	1	1	1	1
5	6	6	1	1	0	0	0	0

[†]The serial number of mice.

Supplementary Table S3 Summary of fatty acid synthase and frizzled class receptor 10 (FZD10) expressions detected by immunohistochemisty in xenografts of CNE1-shRNA-NC cells

No. of mice	FASN		FZD10	
	+	−	+	−
Control	5	0	5	0
Epigallocatechin gallate (30 mg/kg)	2	3	2	3
Radiation (5 Gy)	2	3	2	3
Radiation (5 Gy) and epigallocatechin gallate (30 mg/kg)	1	4	1	4

Supplementary Table S4 The clinical pathological features of the 122 nasopharyngeal carcinoma patients

Clinical pathological features	No.	Percentage (%)
Age (years)		
<60	96	78.7
≥60	26	21.3
Gender		
Male	91	74.6
Female	31	25.4
N classification		
No	18	14.8
Yes	104	85.2
Distant metastasis		
No	120	98.4
Yes	2	1.6

Supplementary Table S5 The correlation between fatty acid synthase (FASN) and frizzled class receptor 10 (FZD10) expression in tumors from nasopharyngeal carcinoma patients

		FASN		Total	*P*
		Negative	Positive		
FZD10	Negative	31 (75.6%)	10 (24.4%)	41	<0.001
	Positive	19 (23.5%)	62 (76.5%)	81	
Total		50	72		

Supplementary Table S6 Univariate and multivariate survival analysis for assessing the effect of fatty acid synthase/frizzled class receptor 10 (FZD10) expression on the overall survival in nasopharyngeal carcinoma patients

Variate	*N*	*N* of events	Survival rate (%)			Multivariate survival analysis		
			30-month	60-month	*P* (KM)	HR	95% CI	*P* (Cox)
FZD10 (+)	81	40	0.728	0.499	0.027	1.949	0.990 to 3.837	0.054[a]
FZD10 (−)	41	11	0.805	0.726				
FASN (+)	72	37	0.708	0.476	0.014	1.969	1.061 to 3.654	0.032[b]
FASN (−)	50	14	0.820	0.716				
FASN (−)FZD10(−)	31	5	0.839	0.839	0.020			0.053[c]
FASN (+)FZD10(−)	10	6	0.700	0.350		4.916	1.409 to 17.147	0.012[d]
FASN (−)FZD10(+)	19	9	0.789	0.521		3.265	1.092 to 9.766	0.034[e]
FASN (+)FZD10(+)	62	31	0.710	0.494		3.337	1.291 to 8.620	0.013[f]

[a]All 122 nasopharyngeal carcinoma (NPC) patients were divided into 2 groups according to FZD10 expression, namely patients with FZD10(+) and patients with FZD10(−). *P* value represented the significant difference of overall survival (OS) rate between these 2 groups. [b]Similarly, *P* value demonstrated the significant difference of OS rate between patients with FASN(+) and patients with FASN(−). [c]Furthermore, we divided 122 NPC patients into 4 groups according to both FZD10 and FASN expressions, namely patients with FASN(−)FZD10 (−), patients with FASN(+)FZD10(−), patients with FASN(−)FZD10(+) and patients with FZD10(−). *P* value represented the significant difference of OS rate among these 4 groups. Additionally, in contrast to FASN(−)FZD(−), OS was obviously worse in FASN(+)FZD10(−), FASN(−)FZD10(+), and FASN(+)FZD10(−), indicated by [d]*P*, [e]*P*, and [f]*P* respectively.

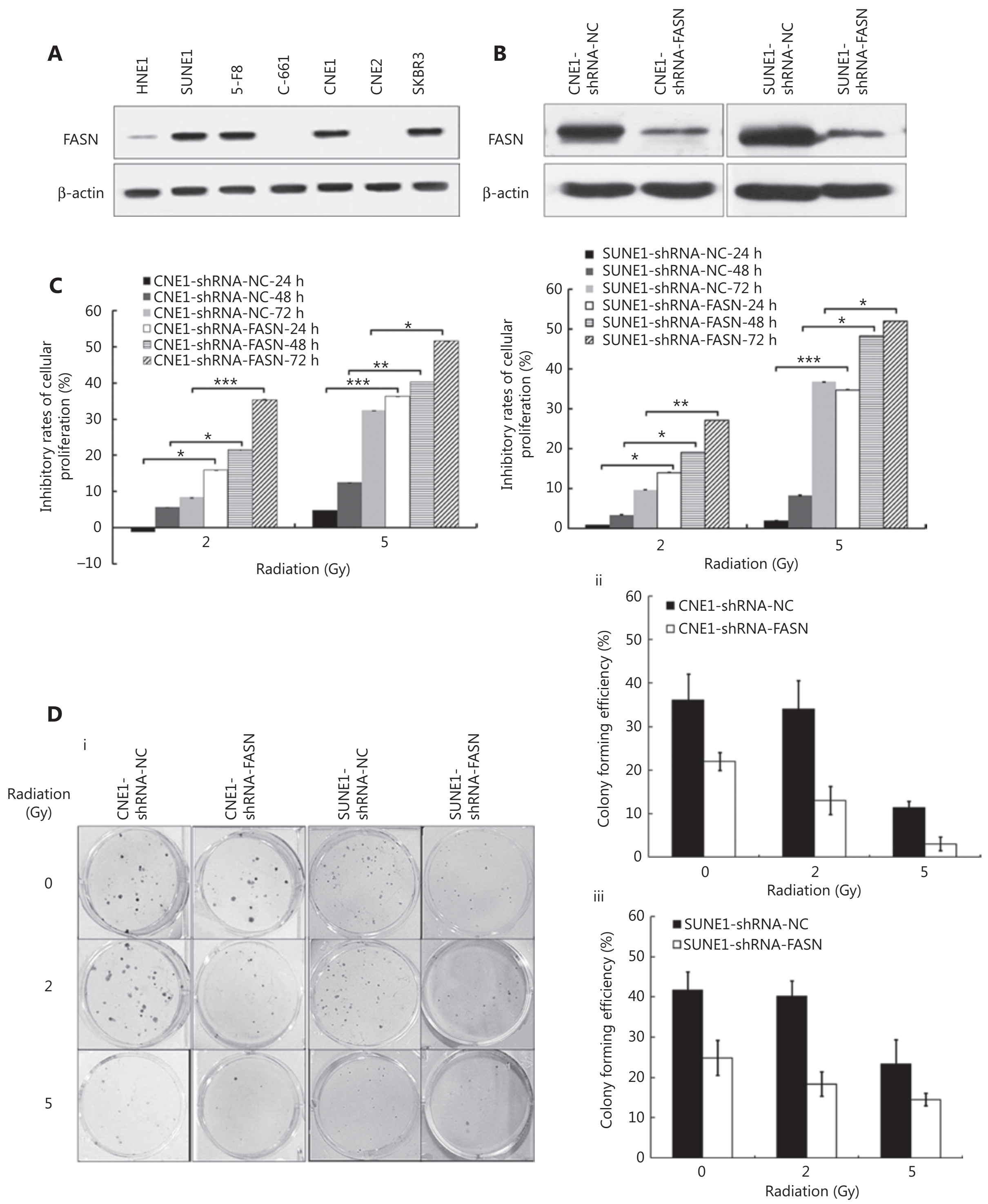

Figure S1 Fatty acid synthase (FASN) knockdown sensitizes nasopharyngeal carcinoma cells to radiation. (A) Expression profile of FASN in different cancer cell lines as determined using WB. β-Actin was used as a loading control. (B) WB analysis of FASN knockdown in CNE1 and SUNE1 cells. (C, D) Effect of FASN knockdown on radiation-induced inhibition of proliferation of CNE1 (left panel) and SUNE1 (right panel) cells as determined using MTT (C) and colony formation (D) assays ($^{*}P < 0.05$; $^{**}P < 0.01$; $^{***}P < 0.001$).

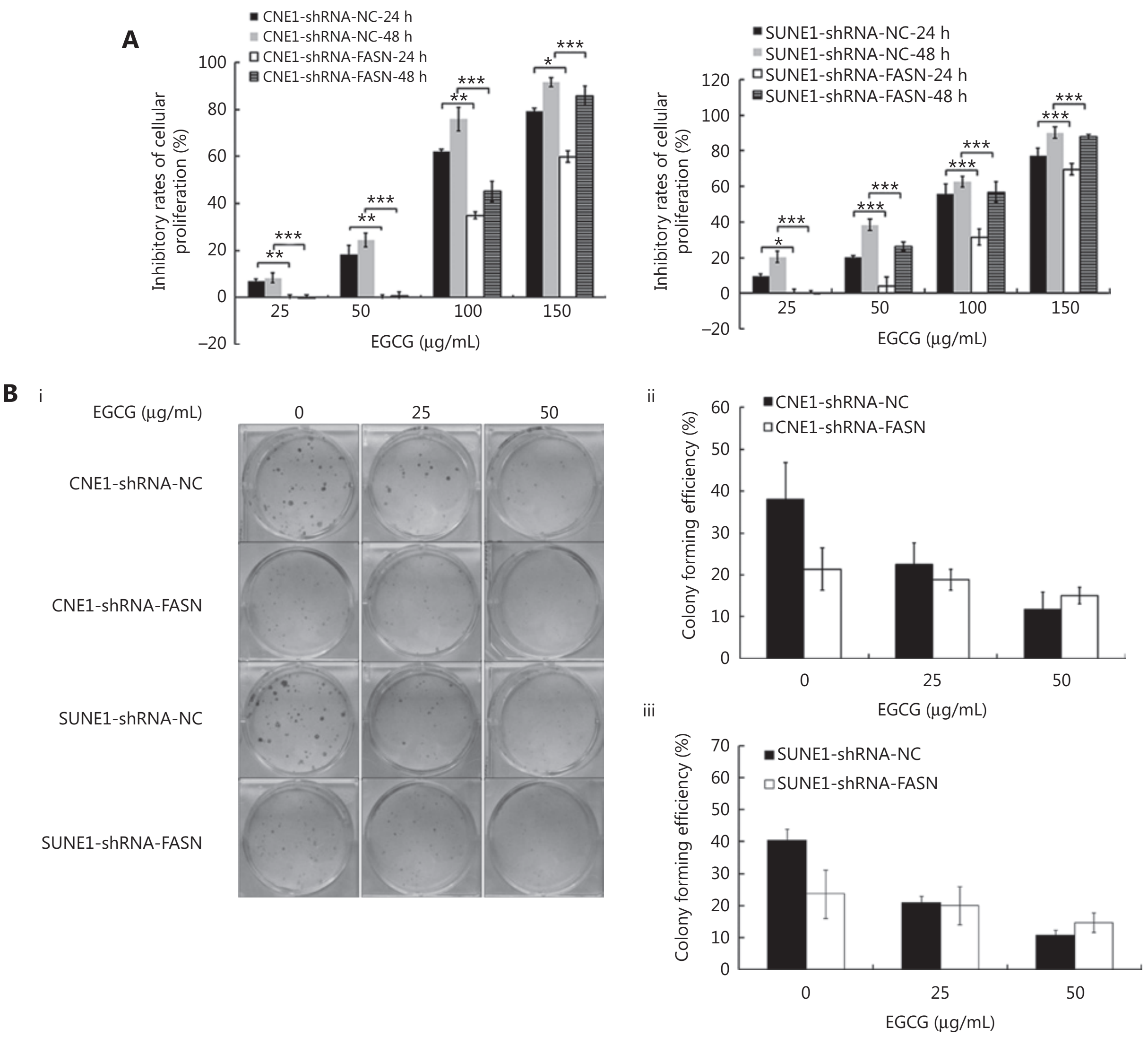

Figure S2 Epigallocatechin gallate at low concentration is selective to fatty acid synthase (FASN) in nasopharyngeal carcinoma cells. The effect of epigallocatechin gallateon proliferation of CNE1 and SUNE1cells following FASN knockdown was tested using the MTT assay (A) and colony formation (B) assays at different concentrations and at different time points. **P* < 0.05; ***P* < 0.01; ****P* < 0.001.
